# A new device to seal large coronary aneurysms: a case report

**DOI:** 10.1186/1752-1947-4-238

**Published:** 2010-08-03

**Authors:** Danzi Battista Gian, Guido Angelo Pomidossi, Filippo Casolo, Marco Centola, Roberto Ferraresi, Chaim Lotan

**Affiliations:** 1 Department of Cardiology, I.R..C.C.S. Ospedale Maggiore Policlinico, Milano, Italy; 2 Heart Institute, Hadassah-Hebrew University Medical Center, Jerusalem, Israel

## Abstract

**Introduction:**

Coronary artery aneurysm is an uncommon disease. It is defined as a coronary artery dilatation, exceeding the diameter of the normal adjacent segment or the diameter of the patient's largest coronary vessel by 1.5 to 2 times. Coronary artery aneurysms are typically diagnosed by coronary angiography. The prognosis of coronary artery aneurysm is not well known and the management is challenging.

**Case presentation:**

A 68-year-old Italian-Caucasian man presented to our hospital with angina. Coronary angiography revealed a large coronary aneurysm of the right coronary artery, which was successfully treated by the percutaneous implantation of an MGuard™stent.

**Conclusion:**

This case report provides evidence that coronary artery aneurysms, even if very large, can be safely treated by MGuard™stent implantation. We strongly emphasize the high flexibility and good deliverability of this device, which leads to the complete exclusion of the aneurysm mediated by the process of endothelization of its thin mesh sleeves.

## Introduction

**Figure 1:**
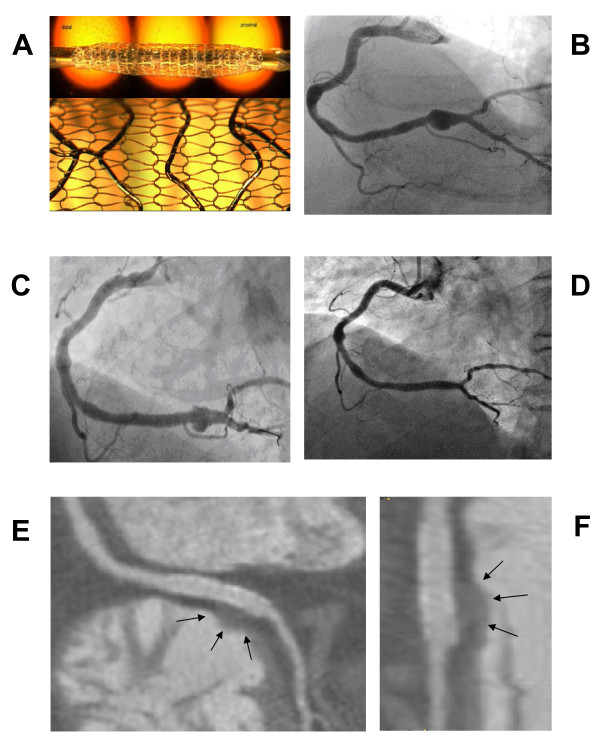
**Coronary angiography and CT scan before and after MGuard stent implantaion**. (A) The unexpanded (top) and the expanded stent covered with a sleeve mesh (bottom). Reproduced with permission from InspireMD. (B) Angiography of the right coronary artery with the presence of a large saccular aneurysm involving the distal part of the artery to the crux cordis. (C) Partial opacification of the aneurysmal sac through the holes of the mesh just after stent implantation. (D) Coronary angiography at one month follow-up showing the exclusion of the aneurysm. (E) Coronary CT scan at one month: multiplanar reformation of the right coronary artery near the crux cordis; on the right ventricle side of the distal part of the stent, is clearly demonstrated the water density remnant of the treated aneurysm (arrows): low density fat is surrounding the proximal stent. (F) Coronary CT scan at one month: The magnified view of the stent allows for a better identification of the treated aneurysm (arrows).

Coronary artery aneurysm is a rare disorder characterized by an abnormal dilatation (diameter >50% of the normal adjacent segments) of a localized or diffuse portion of the coronary artery [1,2]. The detected incidence on angiography varies from 0.3% to 4.9% in large studies [1,2]. Atherosclerosis is the most common etiology estimated at 50% of cases diagnosed in adults; independent of the etiology, the aneurysmal segment produces turbulent blood flow with an increased incidence of myocardial ischemic manifestations (acute coronary syndromes, thromboembolism, rupture or vasospasm) [3-5]. The indication for treatment and the best modalities still need to be defined [6]. In recent years different studies have reported the treatment of coronary aneurysm with the polytetrafluoroethylene (PTFE) covered stents as a valuable alternative to surgery in selected cases (diameter six to 10mm) [7,8]. However, the use of this device is limited by its dimension, by the low flexibility (that makes implantation very difficult in tortuous vessels), by the lack of access of side branches and by the inherent risk of restenosis. The MGuard™stent (InspireMD Ltd, Tel Aviv, Israel) is a novel coronary device composed of a multicellular stainless steel stent with an integrated polyethylene terephthalate (PET) protective sleeve mesh (15 μm single PET knit fiber with 150 μm × 150 μm apertures) (Figure 1a). The sleeve acts as a snowshoe, averaging the expansion impact while preventing embolic dislodgment. This stent has a high flexibility and a low profile that can challenge very complex anatomies; the process of endothelization of this device transforms the mesh sleeve into a membrane that has the potential to seal coronary aneurysms [9,10].

## Case presentation

A 68-year-old Italian-Caucasian man who had a previous angioplasty to an obtuse marginal branch presented to our institution for angina and a positive myocardial perfusion imaging test (reversible perfusion defects on inferior wall). An angiography showed a patent stent on obtuse marginal artery and a large coronary aneurysm of the right coronary artery (6.7 mm in diameter and 15.2 mm in length) (Figure 1b; additional file 1)[1,2]. A 3.5 to 24 mm long MGuard™stent was implanted, using a pressure of 16 bar (additional file 2). After stent deployment, the injection of dye showed partial opacification of the aneurysmal sac through the holes of the net, with some stagnation of blood (Figure 1c; additional file 3). His hospital stay was uneventful and our patient was discharged the day after the procedure with dual anti-platelet therapy. At one month our patient was totally asymptomatic. The coronary computed tomography (CT) scan showed a good stent apposition (Figure 1e,f) with complete exclusion of the aneurysm that was also confirmed by angiography (Figure 1d; additional file 4).

## Conclusions

Coronary artery aneurysms may be complicated by thrombosis and rupture. The optimal treatment options of coronary aneurysms are still largely debated. They include anticoagulation, covered stents, reconstruction, resection and exclusion with bypass [11]. The PTFE-covered stents have been used to treat coronary artery aneurysms, but the restenosis rate and long-term effectiveness of covered stents are unknown. The newly developed balloon-expandable MGuard™stent system with its combination of an ultra-thin polymer mesh sleeve attached to the external of a bare metal stent surface has been designed to provide embolic protection during percutaneous coronary intervention restenosis by bare metal stents, peripheral embolism following stent implantation in vein grafts, and conditions in which there is a discontinuity of the coronary lumen (rupture or perforation, aneurysm, and fistula). This device demonstrated excellent performance in a highly complex lesion subset such as cover coronary artery perforations or arteriovenous fistulas with high success and acceptable rates of acute complications. Based on its technical features, we decided to use the MGuard™stent to seal a coronary artery aneurysm. This experience suggests a new potential indication for the use of MGuard™coronary stent as an attractive alternative treatment with less risk of in-stent restenosis. In human coronary arteries the complete exclusion of the aneurysmal sac (that is mediated by the process of endothelization of the ultra-thin PET mesh sleeve) is not immediate and seems to require at least four weeks. The high flexibility and the good deliverability make the MGuard™device an attractive alternative to the currently available covered stent for an effective treatment of coronary aneurysm.

## Competing interests

GBD certifies that GAP, FC, MC, RF, and he have no financial potential conflicts of interest and no financial relationship with the company that produces the MGuard stent.

The idea of the case report was conceived and executed by GBD, and his team with no financial interest affecting the manuscript. The manuscript was not subject to approval by the company. Minor changes were offered by CL, on a technical level to ensure accuracy. CL, the co-author of the paper, is a consultant for Inspire MD, the company that produces the MGuard stent.

## Authors' contributions

The idea of the case report was conceived and executed by GBD. GBD and GAP interpreted the patient data regarding the cardiac disease. GBD and GAP decided the clinical strategy and were operators during the coronary angiography. They wrote the paper. FC analyzed CT scan images and gave suggestions regarding the images 'selections. MC and RF were major contributors in writing the manuscript. Minor changes regarding the technical features of the device were offered by CL on a scientific level to ensure accuracy. All authors read and approved the final manuscript.

## Consent

Written informed consent was obtained from the patient for publication of this case report and any accompanying images. A copy of the written consent is available for review by the Editor-in-Chief of this journal.

## Supplementary Material

Additional file 1 **Coronary angiography showing a large coronary artery aneurysm**. Right anterior oblique view of the right coronary artery with the presence of a large aneurysm involving the distal part of the artery till the crux cordis. Click here for file

Additional file 2 **MGuard stent implantation**. Stent deployment at a pressure of 16 bar. Click here for file

Additional file 3 **Coronary angiography after stent implantation**. Opacification of the aneurysmal sac through the holes of the net, with some stagnation of blood. Click here for file

Additional file 4 **Coronary angiography at one month follow up**. Follow-up angiography at one month showing the complete exclusion of the aneurysmal sac. Click here for file
